# Ventilator-day reductions not associated with reintubations and further reduced by an early mobilization program

**DOI:** 10.1186/cc14347

**Published:** 2015-03-16

**Authors:** M Palmquist-Tess, A Adams, J Mangan, D Owen, M LeClaire

**Affiliations:** 1 HCMC, Minneapolis, MN, USA

## Introduction

Mechanical ventilation is associated with increased risk of pneumonia, barotrauma, VILI, VAP, ARDS and mortality. From 2009 to 2014 in the MICU/SICU of our facility, efforts to reduce ventilator-days included: noninvasive ventilation, sedation reduction, daily sedation vacations and weaning protocols. In 2013, an early mobilization of ventilated patients in the SICU was initiated. Aggressive ventilator-day reduction efforts may be expected to lead to premature extubations and reintubations.

## Methods

Ventilator-day data were compiled from 2009 to 2014 for MICU and SICU in our facility. Reintubation rates were calculated when intubations were required >1 day after an extubation.

## Results

Ventilator volume ranged from 639 to 766 distinct patients/ year in the MICU and from 555 to 687 for the SICU. Ventilator-day reduction was significant (*P *< 0.01) for the MICU* (7.7 to 5.5, -29%) and the SICU* (5.91 to 5.20, -12%). Reduction patterns differed between the units as the SICU had a distinct reduction (**P = 0.007) between 2012 and 2013 coinciding with implementation of an early mobilization program. Reintubation rates differed between the units and rates did not increase with decreasing mean patient ventilator-days. See Table 1.

**Table 1:** Mean ventilator-days/reintubation rates.

	2009	2010	2011	2012	2013	2014
MICU mean ventilator-days	7.71	6.81	5.90	5.88	5.50	5.50*
MICU reintubation rates	14.5%	9.8%	9.7%	9.8%	13.9%	8.3%
SICU mean ventilator-days	5.91	5.93	5.68	5.93	4.93**	5.20*
SICU reintubation rates	5.2%	3.0%	4.1%	4.3%	4.7%	7.5%

*, **Significance as described in Results.

## Conclusion

Initiatives to reduce ventilator-days per patient realized significant reductions from 2009 to 2014 while reintubation rates were unaffected. One component of the bundle, early mobilization, introduced in the SICU in 2013 was associated with an additional reduction in mean ventilator-days.

